# Automatic distractor generation for multiple-choice English vocabulary questions

**DOI:** 10.1186/s41039-018-0082-z

**Published:** 2018-10-01

**Authors:** Yuni Susanti, Takenobu Tokunaga, Hitoshi Nishikawa, Hiroyuki Obari

**Affiliations:** 1 Department of Computer Science (W8E-6F), Tokyo Institute of Technology 2-12-1 Oookayama, Meguro-ku, Tokyo, 152-8552 Japan; 2 College of Economics, Aoyama Gakuin University, Tokyo, Japan

**Keywords:** Distractor, English vocabulary question, Automatic distractor generation, Multiple-choice question

## Abstract

The use of automated systems in second-language learning could substantially reduce the workload of human teachers and test creators. This study proposes a novel method for automatically generating distractors for multiple-choice English vocabulary questions. The proposed method introduces new sources for collecting distractor candidates and utilises semantic similarity and collocation information when ranking the collected candidates. We evaluated the proposed method by administering the questions to real English learners. We further asked an expert to judge the quality of the distractors generated by the proposed method, a baseline method and humans. The results show that the proposed method produces fewer problematic distractors than the baseline method. Furthermore, the generated distractors have a quality that is comparable with that of human-made distractors.

## Introduction

Recent advances in natural language processing (NLP) have enabled us to build more advanced applications in the educational field, especially in learning and testing. They include the utilisation of NLP technologies and language resources for automating student assessment, instruction and curriculum design. Among others, applying NLP to second-language learning has attracted extensive attention, including automated essay scoring (Shermis and Burstein 2003) and automatic question generation (Araki et al. 2016; Hoshino and Nakagawa 2005; Pino and Eskenazi 2009; Satria and Tokunaga 2017; Sumita et al. 2005; Susanti et al. 2015). The use of fully automated systems in second-language learning could significantly reduce the burden on human experts to teach students, create tests and evaluate the development of student’s abilities. At the very least, they could assist the human experts with these teaching activities. Automated systems are also useful for the self-study for students. For instance, students would be able to work on questions automatically generated by the system according to their current ability.

**Fig. 1:**
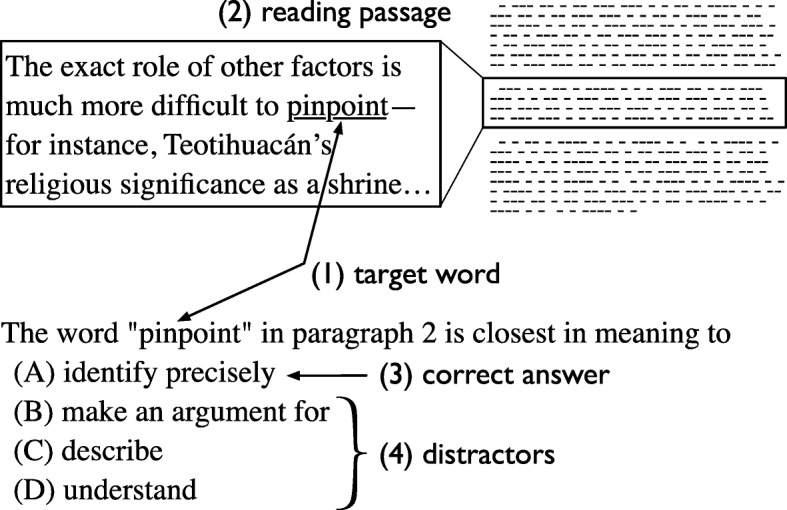
Closest-in-meaning vocabulary question (source: a TOEFL iBT question from past test, taken from the official website, www.ets.org.)

Standardised English language tests such as Test of English as a Foreign Language (TOEFL), Test of English for International Communication (TOEIC) and International English Language Testing System (IELTS) often use a multiple-choice format (as depicted in Fig. 1) because of its efficiency in scoring. However, the difficulty and cost of developing multiple-choice questions have been the primary challenge of this format (Downing 2006). Because of this, research on automatic question generation, especially on multiple-choice question generation, has attracted much attention recently. Various resources and technologies in NLP can potentially contribute to the automatic generation of multiple-choice questions, as has been done by Mitkov and Ha (2003) who used the WordNet (Fellbaum 1998) as a lexical dictionary resource. More recently, Susanti et al. (2015) also used WordNet to generate multiple-choice English vocabulary questions. Another study was performed by Araki et al. (2016), who applied semantic analysis to texts for generating multiple-choice questions for English language learners.

Rodriguez (2005) stated that the quality of multiple-choice questions relies heavily on the quality of their options. His claim is supported by Hoshino (2013), who noted that test takers tended to employ a choice-oriented strategy when working on multiple-choice questions. Therefore, the quality of the question options, especially the distractors (wrong options), affects the quality of the question, as inappropriate distractors enable the test takers to guess the answer easily (Moser et al. 2012) or cause them to unnecessary confusion.

Nevertheless, few studies on automatic question generation have focused on distractor generation. Haladyna (2004) pointed out that generating distractors was the most difficult part of multiple-choice question generation. As in the manual writing of questions, developing appropriate distractors remains a difficult task in automatic question generation. Some studies have generated distractors for fill-in-the-blank language questions using simple techniques such as random selection from words in the same document (Hoshino and Nakagawa 2005), employing a thesaurus (Sumita et al. 2005) and collecting similar candidates of the target word in terms of their frequency and dictionary-based collocation (Liu et al. 2005).

Other studies have employed more advanced techniques and resources for distractor generation, mostly for the fill-in-the-blank English vocabulary questions. For instance, Pino and Eskenazi (2009) and Correia et al. (2010) used graphemic (morphological and orthographic) and phonetic variants of the target word as distractor candidates. Correia et al. (2010) employed lexical resources to filter distractor candidates considering the target word’s synonym, hyponym and hypernym. Sakaguchi et al. (2013) utilised common learner errors that were constructed from error-correction pairs on a language learning site, Lang-8^1^. In each pair of corrections, the error was a candidate distractor for the target word. Zesch and Melamud (2014) applied context-sensitive lexical inference rules to generate verb distractors that are not semantically similar to the target in the fill-in-the-blank context but might be similar in another context. More recently, Jiang and Lee (2017) proposed the use of a semantic similarity measure based on the word2vec model (Mikolov et al. 2013) for generating plausible distractors of Chinese fill-in-the-blank vocabulary questions.

The present study focuses on generating distractors for English vocabulary questions as used in TOEFL. Figure 1 illustrates an example of the type of question we discuss in this study. The vocabulary questions in TOEFL have a distinct characteristic; instead of asking for the best word to fill the gap as in the fill-in-the-blank type questions, they ask for the closest-in-meaning word of a target word (the vocabulary being asked in a question) used in a reading passage. However, the result of this study can contribute to the fill-in-the-blank questions as well because both types of questions share the same requirements for their distractors.

We conducted two evaluations: (1) a test taker-based evaluation and (2) an expert-based evaluation. In the first evaluation, we asked English learners to complete a set of question that differed only with respect to the distractor set. Otherwise, they shared the same reading passage and correct answer taken from a human-made question item. Each distractor set contains three distractors that are either human-made, generated by the baseline method or generated by the proposed method. We evaluated the quality of the distractors from the test takers’ responses by applying Neural Test Theory (Shojima et al. 2008), which is a test theory for analysing test results that grades the test takers into several ranks. According to Susanti et al. (2017), this test is useful for evaluating the statistical characteristics of options in multiple-choice questions. In the expert-based evaluation, we asked a professional item writer to evaluate the same sets of distractors as in the test taker-based evaluation. We also discuss how the two evaluation results relate.

The contributions of the present study are as follows:

1. We proposed a method of distractor generation for multiple-choice vocabulary questions that is superior to the state-of-the-art method.
2. We thoroughly analysed the evaluation results from two different perspectives, i.e. the test taker-based and the expert-based evaluations.

The next section (“Methods” section) describes the distractor generation including the proposed method and the state-of-the-art method used as the baseline. The evaluation design is presented in the “Evaluation design” section. The “Results and discussion” section discusses the result and analysis of the evaluation and is followed by the conclusion and future work directions in the “Conclusion” section.

## Methods

In this study, we implemented a distractor generation method introduced by Jiang and Lee (2017) as a baseline because their work is the latest state-of-the-art method that targets the most similar task to the current study. Although their method generates Chinese fill-in-the-blank vocabulary questions, the method is independent of the language because it takes a corpus-based approach. We can hence adapt the method for English by replacing the corpus. Another difference is the question type to generate, i.e. fill-in-the-blank questions versus closest-in-meaning questions. These questions differ in whether the target word is present in the options as a correct answer (fill-in-the-blank) or present in the reading passage (closest-in-meaning). There is no difference in the characteristics of the distractors in both types of vocabulary questions.

In the following, we describe the baseline in detail, followed by our proposed method. We then compare the two methods as summarised in Table 1. For all methods, distractor generation consists of three steps: (1) distractor candidate collection, (2) distractor candidate filtering and (3) distractor candidate ranking.

**Table 1:** Methods to be compared

Step	Baseline (Jiang and Lee 2017)	Proposed
Selection	All words in English	(1) Synonym of co-occurrence words in the passage
	Wiki Corpus with the	(2) Sibling words in the taxonomy and synonyms of
	same part of speech as	synonyms
	the target word	(3) Words in the JACET8000 list with the close level
		to the correct answer
Filtering	Trigram filtering	Criteria by (Heaton 1989) and synonym filtering
Ranking	word2vec-based semantic	GloVe-based semantic similarity between the target
	Similarity between the	word and a distractor candidate, and between the
	target word and a	correct answer and a distractor candidate, and word
	distractor candidate	collocation

### Baseline method

#### Distractor candidate collection and ranking

To collect distractor candidates, Jiang and Lee (2017) extracted all the words in the Chinese Wiki corpus and ranked them on the basis of their various similarity criteria to the target word. The similarity criteria consist of the word difficulty level (frequency-based) similarity, spelling similarity, PMI-based word co-occurrence with the target word and word2vec-based word similarity. They ranked the candidates according to each criterion and evaluated the results. Their evaluation showed that the word2vec-based criterion outperformed the others; thus, in this study, we implemented this criterion for collecting the distractor candidates.

Jiang and Lee (2017) trained a word2vec model on the Chinese Wiki corpus^2^. Because we adapted their method for English vocabulary questions, we used a word2vec model pre-trained on English Wikipedia^3^.

#### Distractor candidate filtering

Jiang and Lee (2017) filtered the ranked distractor candidates to remove candidates that are also considered to be an acceptable answer. They examined whether the distractor candidates collocate with the words in the rest of the carrier sentence^4^, by filtering based on the trigram and dependency relations.

- Trigram filtering: the trigram is formed from the distractor candidate and its two adjacent words (the previous and following words) in the carrier sentence. We implemented this filtering without modification in our implementation for English vocabulary questions.
- Dependency relation filtering: the implementation by Jiang and Lee (2017) considers all the dependency relations with the distractor as a head or child. We implemented this filtering with a small corpus^5^, but the filtering did not remove any candidates. Hence, we decided not to implement this filtering.

The three highest ranked candidates after the filtering were chosen as the final distractors for the question.

### Proposed method

#### Distractor candidate collection

We collected the distractor candidates from two main sources that reflect two different relations with the target word. The first source is synonyms of the words in the reading passage that have the same part of speech and tense as the target word, with an assumption that those words share the same topic of the reading passage. The second source is siblings of the target word in the WordNet taxonomy. Because siblings share the same hypernym, the siblings of the target word should share a similar meaning but also have a certain difference in meaning.

In addition to these two sources of distractor candidates, we utilise the JACET8000 word list (Ishikawa et al. 2003) as the third source of distractor candidates. JACET8000 is a word list designed for Japanese English learners. It ranks 8000 basic English words according to their frequency in the British National Corpus^6^ supplemented with six million tokens of texts targeted at the needs of Japanese students. The 8000 words are divided into eight groups of 1000 words; each group corresponds to their level of word difficulty with level 1 being the easiest.

We consider JACET8000 suitable for generating English vocabulary questions because it has been compiled for the purpose of English learning. Our observations tell us that most distractors in human-made vocabulary questions have the same or almost the same level of difficulty as the correct answer. Thus, as the distractor candidates, the present study utilises the words in the JACET8000 word list for which the level differs at most by two levels from that of the correct answer. For example, if the correct answer is level 4, the distractor candidates are collected from the words of levels 2–6.

Furthermore, to top up insufficient distractor candidates from WordNet, we also add the synonyms of synonyms and words related to the target word according to the *Merriam-Webster Dictionary*.

#### Distractor candidate filtering

The collected distractor candidates are further filtered following English vocabulary questions writing guidelines (Heaton 1989), which are summarised below.

1. Question options should have the same part of speech as the target word.
2. Distractors should have a word difficulty level that is similar to that of the correct answer.
3. Question options should have approximately the same length.
4. A pair of synonyms in the question options should be avoided.
5. Antonyms of the correct answer should be avoided as distractors.
6. Distractors should be related to the correct answer, or come from the same general topic.

Vocabulary questions in the present study ask for the word closest-in-meaning to the target word. Thus, the distractors must not have the same or a very similar meaning to either the target word or the correct answer. To guarantee that the distractors are not synonyms of the target word, we filter out synonymous candidates using the synonym list from WordNet and the *Merriam-Webster Dictionary* in addition to the criteria specified by Heaton (1989).

#### Distractor candidate ranking

Although the distractors must have a different meaning from both the target word and the correct answer, they must also be able to distract the test takers from the correct answer. Because the present study focuses on the closest-in-meaning vocabulary question, distracting distractors should be similar to the target word or correct answer in some respects. Unlike fill-in-the-blank questions, where the target word and correct answer are the same, in the closest-in-meaning questions, we can utilise both the target word and correct answer to generate distractors so that the distractors are semantically close to the target word but far from the correct answer. To rank distractor candidates, the baseline adopts a word embedding-based semantic similarity measure. In contrast, the present study introduces a new ranking metric *r*(*c*) that aggregates word embedding-based semantic similarity and word collocation information for ranking the distractor candidates *c* with respect to the target word (*tw*), reading passage (*rp*) and correct answer (*ca*), which is given by:

1 $$ r(c) = \text{rank}(\text{sim}(c,tw))+\text{rank}(\text{col}(c,rp))-\text{rank}(\text{sim}(c,ca))  $$

where sim(*w*_*i*_,*w*_*j*_) is the semantic similarity between words *w*_*i*_ and *w*_*j*_; col(*w*_*i*_,context) is a collocation measure of word *w*_*i*_ and its adjacent two words in the given context, and rank(*f*(·)) returns the rank of the value of *f*(·) in descending order. We use ranks instead of their raw scores because they are easier to integrate into a single score.

To calculate sim(*w*_*i*_,*w*_*j*_), the present study employs the cosine similarity of the word vectors derived by the word embedding GloVe algorithm rather than word2vec because it is more efficient (Pennington et al. 2014). We used the pre-trained GloVe word vectors^7^. We calculate the collocation measure col(*w*_*i*_,context) on the basis of the frequencies of two bigrams: (*w*_*i*−1_,*w*_*i*_) and (*w*_*i*_,*w*_*i*+1_) in the context. The bigram statistics were generated using the module provided by the NLTK Python Package^8^ and the English Text corpora in the same package^9^.

The idea behind Eq. (1) is that we want to obtain a distractor candidate *c* that is similar to target word *tw* (a large sim(*c*,*tw*), i.e. has a high rank(sim(*c*,*tw*))), and frequently collocates with the adjacent words in the reading passage *rp* (a large col(*c*,*rp*), i.e. has a high rank(col(*c*,*rp*))), but is not similar to the correct answer *ca* (a small sim(*c*,*ca*), i.e. has a low rank(sim(*c*,*ca*))). Thus, we prefer distractor candidates with a smaller value of *r*(*c*).

### Evaluation design

#### Question data

We selected 45 target words (TW 1–45) from real closest-in-meaning vocabulary questions collected from the ETS official site^10^ and preparation books of TOEFL iBT, which are published by the official TOEFL organisation^11^. The selection was made such that the part of speech categories of the target words were balanced.

For each question, we used the three human-made distractors in the original TOEFL question as a reference distractor set. We then determined two additional sets of three distractors using the baseline and proposed methods. For each automatically generated method, the set of three distractors was made by selecting from the top three candidates in the ranked candidate list of that method. The original reading passage and correct answer were used to automatically generate the distractors. In total, we prepared 135 question items with 45 items each set. The order of the distractors was randomised in each question.

We conducted two evaluations, test taker-based and expert-based evaluations; they are explained in the following sections.

#### Test taker-based evaluation

The aim of this evaluation is to evaluate the validity of the distractor candidates when they are used in a real test setting. We administered the question set described above to English learners and evaluated the quality of the distractors based on their responses. We used a Latin square design to design the question sets, as shown in Table 2. For instance, in question set QS.A, the distractor sets for target words (TWs) 1 to 15 are generated by the baseline method and TWs 16 to 30 by the proposed method and TWs 31 to 45 are the original TOEFL distractors created by humans.

**Table 2:** Configuration of the question sets

Student group	Number of students	Question set	Baseline	Proposed	TOEFL
G1	19	QS.A	TW 1–15	TW 16–30	TW 31–45
G2	23	QS.B	TW 16–30	TW 31–45	TW 1–15
G3	38	QS.C	TW 31–45	TW 1–15	TW 16–30

##### Participants

A total of 80 Japanese university undergraduate students participated in the experiment. We divided them into three student groups, G1, G2 and G3, according to their school class and administered a different question set to each student group. Table 2 shows the assignment of the question sets to the student groups.

##### Experimental procedure

The experiment was conducted in the form of an online test. The participants completed the test using their own computer, but each group worked on the question set together in the same classroom. The experiment comprised three sessions. In each session, one of the three groups worked on their assigned question set. A session lasted roughly 30–40 min.

#### Expert-based evaluation

The aim of this evaluation is to evaluate the quality of the automatically generated distractors using a human expert. Because of limited resources, we asked one human expert to evaluate the questions. However, we believe his judgement is reliable because he is an experienced professional writer of these questions.

We provided the expert with an evaluation guideline that includes the question writing guidelines presented in the “Distractor candidate filtering” section. Given a target word and its corresponding reading passage, the expert evaluated each of the three distractor sets used in the test taker-based evaluation by giving it a score of 1–5, where 1 indicates very low quality and 5 indicates very high quality. We also provided an optional “comment” field where he could write any possible reasons for giving a low score to a set of distractors, or explain why distractors were problematic, if any existed in the set.

## Results and discussion

### Test taker-based evaluation

#### Correlation with test takers’ proficiency scores

We calculate the correlation between test takers’ scores on the questions and their TOEIC scores, which we treated as the ground truth proficiency scores. The idea is that if the test takers’ scores on the machine-made questions show a strong correlation with their TOEIC scores, then the machine-made questions are able to measure the test taker’s proficiency. Table 3 presents the Pearson correlation coefficients between the scores.

**Table 3:** Pearson correlation coefficients between test scores (averaged for all groups)

Test scores	Baseline	Proposed	TOEFL
TOEIC	0.290	0.290	0.342
TOEFL	0.302	0.425	1.000

All *p* values are less than 0.05

The scores on the questions generated by both automatic methods show a lower correlation with their TOEIC scores than those of the original TOEFL questions. However, all methods indicate a low correlation in absolute terms. This is because the TOEIC score reflects various kinds of English proficiency of the test takers, whereas the generated questions concern only their vocabulary.

Focusing on the vocabulary ability, we also calculated the correlation coefficients between test takers’ scores on the machine-generated questions and those of the original TOEFL questions. This yielded positive correlations with coefficients of 0.425 (*t* = 5.039, df = 38, *p* value < 0.05) for the proposed method and 0.302 (*t* = 6.08, df = 37, *p* value < 0.05) for the baseline. (Evans 1996) categorised a correlation coefficient of 0.425 as “moderate” and 0.302 as “weak” correlation. This result is encouraging because it indicates that questions using the proposed method are more successful than those created using the baseline at measuring the test takers’ proficiency with respect to the original TOEFL questions.

#### Neural Test Theory analysis

Neural Test Theory (NTT) (Shojima 2007) is a test theory for analysing test data that grades the test takers into several ranks (on an ordinal scale). The idea behind this theory is that a test cannot distinguish test takers who have nearly equal abilities; the most that a test can do is to grade them into ranks. Susanti et al. (2017) applied the nominal neural test (NNT) model (Shojima et al. 2008), which is a variant of NTT for nominal-polytomous data, which is suitable for our vocabulary multiple-choice questions. NTT is useful for evaluating the statistical characteristics of options in multiple-choice questions. The item category reference profile (Shojima et al. 2008), ICRP for short, is a feature of NNT representing the probability that the test taker in a certain rank selects a certain question option in their responses to a certain question. Susanti et al. (2017) claimed that ICRP can be used to clarify the validity of the question options because it shows how test takers at each rank behave against each option of the question. For instance, ICRP can be used to clarify whether a distractor correctly deceives the low-ranked test takers compared with the high-ranked test takers.

**Fig. 2:**
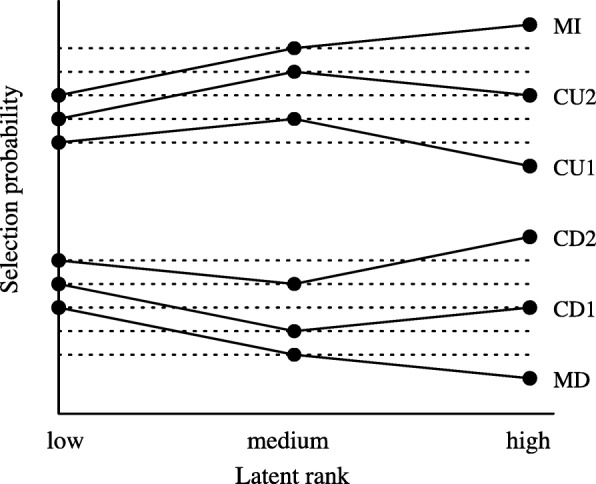
ICRP categories (image source: Susanti et al. (2017))

Susanti et al. (2017) further categorised the ICRP into six categories based on the magnitude of the relations between the probability that the option is selected by test takers in the corresponding student rank, as shown in Fig. 2. According to Susanti et al. (2017), the MD options are most favourable for distractors because the role of a distractor is to deceive a test taker into selecting it instead of the correct answer. Hence, the options that tend to be more selected by the lower-ranked test takers are good distractors. Such options should show a decreasing curve similar to the MD options in Fig. 2. They further claimed that the MD options are the best for distractors, followed by the CU1 and CD1 options, then the CU2 and CD2 options. The MI options are the worst options for distractors.

In the present study, we applied the NNT model to our student response data, following the settings of Susanti et al. (2017), and counted the number of distractors in each ICRP category for each method as shown in Table 4.

**Table 4:** ICRPs of the distractors for each method

	Baseline	Proposed	TOEFL
MI	40	26	24
CU2	6	8	5
CD2	13	6	9
CU1	10	13	12
CD1	9	15	18
MD	46	51	50

The three methods produced more or less a similar number of the favourable MD distractors. However, as shown in the first row of Table 4, the proposed method produces fewer MI distractors (least favourable category for a distractor) than the baseline. The original TOEFL questions, as expected, produced the smallest number of MI distractors. This result is encouraging because it shows that the proposed method succeeded in removing the problematic distractor candidates better than the baseline.

We further analysed the MI distractors to find the reasons these distractors were categorised as MI distractors. The probability of choosing the MI distractors increases as the the test taker’s rank increases. This indicates that more high-proficiency test takers are deceived by this distractor than low-proficiency test takers. Knowing the reasons helps us to understand the behaviour of each method when producing those distractors. We found that MI distractors could be classified into the following four categories. SYN The distractor is a synonym of the target word or correct answer, e.g. the distractor “support” for the target word “assistance”, where the correct answer is “help”. We looked up two dictionaries^12^, and if the distractors are listed as a synonym in one of the dictionaries, we classified them in this category. This type of distractors is not appropriate for use in tests. CON This distractor can be replaced in the given context, e.g. the distractor “move” for the target word “cope” when the correct answer is “adapt” in the following context “…dinosaurs were left too crippled to *cope*, especially if, as some scientists believe …”. In this example, the distractor “move” is neither similar to the target word nor the correct answer, but it fits in the context even though it results in a different sentence meaning. We checked the collocation of these words by querying Google search with a distractor and the word it is adjacent to in the reading passage as the query. This kind of distractor is reasonable because the test takers sometimes try to select the option that best replaces the target word in the reading passage. REL This distractor is defined as a word related to the target word or correct answer in a dictionary^13^, e.g. the distractor “storm” is defined as a related word of the target word “bombard” in the *Merriam-Webster Dictionary*. This kind of distractor is also reasonable. UNK This type of distractor has an MI curve in Fig. 2 without any convincing explanation such as one of the above three categories. These distractors can be safely used as a distractor although they are not very distracting.

Table 5 presents the number of the MI distractors categorised according to the above reasons. The results in Table 5 suggest the following conclusions.

**Table 5:** Categorisation of MI distractors

	Baseline	Proposed	TOEFL
SYN	11	0	0
CON	11	13	23
REL	2	1	0
UNK	16	12	1

1. CON is the principal reason for the MI distractors across all methods.

2. On the basis of the above categorisation, the SYN candidates should be rejected as distractors because they are potentially dangerous. None of the MI distractors from the original TOEFL questions and proposed method belong to this category, whereas 11 out of 40 MI distractors of the baseline do and should be rejected. These results indicate that the proposed method succeeded in filtering the problematic candidates in this SYN category.

3. The CON and REL distractors are considered to be reasonable distractors, even though they are MI distractors. According to Table 5, 23 out of 24 original TOEFL distractors belong to this category. The proposed and baseline methods respectively made 14 out of 26 (54%) and 13 out of 40 (33%) reasonable distractors in the CON and REL categories. This result is encouraging because more than half of the MI distractors generated by the proposed method are distracting distractors.

### Expert-based evaluation

We calculate the average judgement scores for all 45 test questions, and the result is as follows: 2.867 (SD 1.471) for the baseline, 4.333 (SD 0.977) for the proposed method and 4.444 (SD 0.840) for the original TOEFL distractors. These average scores indicate that the distractors generated by the proposed method have better quality than those generated by the baseline and comparable quality with respect to the original TOEFL distractors.

The human expert also wrote in a total of 135 comments for all questions. As explained in the “Expert-based evaluation” section, the comments were specifically given to low-scored distractors; in other words, the distractors with comments were the problematic distractors according to the human expert. In total, 71 distractors from the baseline had comments, followed by 39 distractors from the proposed method, and 25 distractors from the original TOEFL distractors. This result is encouraging because the proposed method produced fewer of problematic distractors than the baseline. We grouped the comments into the seven categories presented in Table 6 along with the number of distractors in each category for each method. Note that a distractor can belong to more than one category, so the row “total number of problematic distractors” is not necessarily the sum of the distractors in all categories. A description of the seven categories follows.

**Table 6:** Categorisation of problematic distractors by human expert

	Baseline	Proposed	TOEFL
Too similar with the correct answer or the target word	26	3	1
Different word class	12	10	10
No relation to the correct answer	0	2	0
Different word difficulty	34	22	11
Antonym of the correct answer	3	0	1
Synonym pair	10	0	0
Others	0	3	2
Total problematic distractors	71	39	25

1. *Too similar to the correct answer or the target word*. Comments explicitly state that a distractor is too similar to the correct answer, e.g. “the distractor ‘overcome’ is too close to the correct answer or ‘refined’ is too similar to the correct answer”
2. *Different word class*. Comments concern the difference in the word classes of the correct answer/target word and distractors, e.g. “the distractor ‘arise’ is an intransitive verb, ‘digging out’ is a verb phrase while ‘extending’ and ‘destroying’ are not.”
3. *No relation to the correct answer*. Comments concern criterion 6 in the “Distractor candidate filtering” section, e.g. “the distractor ‘battlefield’ is not related to the correct answer.”
4. *Different word difficulty*. Comments concern criterion 2 in the “Distractor candidate filtering” section, e.g. “all the distractors are much more difficult than the correct answer.”
5. *Antonym of the correct answer*. Comments concern criterion 5 in the “Distractor candidate filtering” section, e.g. “the distractor ‘separate’ is an antonym of the correct answer.”
6. *Synonym pair*. Comments concern criterion 4 in the “Distractor candidate filtering” section, e.g. “the distractor ‘repel’ and ‘repulse’ are synonyms.”
7. *Others*. Comments that are not classified into the above categories, e.g. “the distractor ‘financially rewarding’ should be changed, because it involves the same word as the correct answer.”

Comment category 1 is the severest category because if a distractor is too similar to either the correct answer or the target word, it makes the question invalid because it has more than one correct answer. In this respect, our result is encouraging because the proposed method generated fewer invalid questions in this category. The other comment categories are considered not to be as severe because they do not affect the validity of the question.

We calculated the correlation of the expert scores of the distractor sets. The correlation coefficients are 0.313 (statistically significant at *p* value ≤ 0.05) for the proposed method and human pair and 0.012 (not statistically significant) for the baseline and human pair. This indicates that the expert tends to give similar scores to the proposed method’s distractors and the original distractors. Hence, the distractors generated by the proposed method look more similar to the human-made distractors than those generated by the baseline method from the expert’s point of view.

### Comparison of the expert and test taker-based results

As previously stated, the distractors with comments from the human expert are potentially problematic. We further analysed the behaviour of the distractors in each comment category when they were used in the real test, i.e. in the test taker-based evaluation in the “Test taker-based evaluation” section. We analysed only the responses of the high-proficiency test takers because it is important to determine why high-proficiency test takers were deceived by the problematic distractors. We summarise the results in the following.

1. *Too similar to the correct answer or the target word*. In six out of 30 distractors, no test takers selected the distractor in this category, whereas an average 30% of the high-proficiency test takers selected the other 24 distractors. The distractors in this category must be verified by human experts before they are used in a real test because there is a chance that they are actually the correct answers. One example is the distractor “notion” in a question with the target word “concept” and the correct answer “idea”. In this example, “notion” is a synonym of both the target word and correct answer. Out of 19 test takers, 8 test takers chose the distractor “notion”, whereas only 2 test takers chose the correct answer “idea”. Those 8 test takers did not necessarily choose the wrong answer because the distractor “notion” was indeed correct. This supports the claim that a question should not have distractors with a meaning that is too similar to either the target word or the correct answer.
2. *Different word class*. Less than 23% of the high-proficiency test takers selected 29 out of 32 distractors in this category. Although these distractors are not necessarily problematic, they are not very distracting.
3. *No relation to the correct answer*. Less than 30% of the high-proficiency test takers selected these distractors. As above, although these distractors are not necessarily problematic, they are not very distracting.
4. *Different word difficulty*. The distractors that are easier or more difficult than the other options will stand out and might not be selected by the test takers because their difficulty looks salient. This is supported by the fact that 59 out of 67 distractors in this category were selected by less than 30% of the high-proficiency test takers. Hence, the distractors in this category are not distracting distractors.
5. *Antonym of the correct answer*. More than 50% of the high-proficiency test takers did not select three out of four distractors in this category. If the distractor and correct answer are antonym pair, this suggests that one of them is wrong. This kind of distractor is not distracting.
6. *Synonym pair*. No high-proficiency test takers selected the distractors in this category in four out of ten questions. This is most likely because they found out that a synonym pair in the options could not be a correct answer because a question has only a single correct answer. The distractors in this category should be verified by a human expert before they are used in a real test. One example is the distractors “life-sized” and “lifelike” for a question with the target word “miniature” and the correct answer “small”. No test taker out of 23 chose neither “life-sized” nor “lifelike”. The test takers probably figured out that they were synonym pair, so both could not be the correct answer. This gives evidence that there should not be a synonym pair in the options because the test taker can easily rule them out as a correct answer.

We are also interested in how the problematic distractors from the test taker-based evaluation (the MI distractors) were evaluated by the human expert. The MI distractors are considered problematic because the probability of choosing this distractor increases as the proficiency of the test takers increases. This indicates that more high-proficiency test takers are deceived by this distractor than low-proficiency test takers. Table 7 shows the intersection of the MI distractors in the test taker-based evaluation and the commented distractors by the human expert, which is categorised according to Table 6. Again, because a distractor can belong to more than one category, the sum of distractors in all categories can be larger than the “intersection” row.

**Table 7:** Categorisation of problematic distractors (combined)

Problematic distractor	Baseline	Proposed	TOEFL
Expert-based	82	38	25
Test taker-based	40	26	24
Intersection	24	9	5
Too similar with the correct answer or the target word	9	1	0
Different word class	3	2	3
No relation to the correct answer	0	0	0
Different word difficulty	16	5	1
Antonym of the correct answer	1	0	0
Synonym pair	1	0	0
Others	0	1	1

Table 7 shows that 60% (24 out of 40) of the MI distractors in the baseline were also considered problematic by the human expert. This indicates that the baseline distractors that were judged as problematic by the human expert also behaved inappropriately in the real test. However, the same conclusion could not be drawn from the MI distractors generated by the proposed method and from the original TOEFL questions. Only 35% (9 out of 26) and 21% (5 out of 24) distractors generated by the proposed method and those from the original TOEFL questions, respectively, were judged as problematic by the human expert. This is an encouraging result because those distractors, despite their low score given by the human expert, may still be used in a real test, i.e. the problem is not severe.

## Conclusion

We have presented a novel method for generating distractors for multiple-choice English vocabulary questions. The quality of distractors directly influences the quality of the question because inappropriate distractors allow the test takers to either guess the correct answer easily or unnecessarily confuse them.

The proposed method extends the state-of-the-art method by introducing a new metric for ranking distractor candidates. The new metric aggregates both semantic similarity and word collocation information. The idea is to find distractors that are (1) close to the target word but far from the correct answer in their meaning and (2) collocated with the adjacent words in the given context.

We conducted two evaluations for assessing the quality of the generated distractors: (1) test taker-based evaluation and (2) expert-based evaluation. We prepared 45 questions where the original reading passages and correct answers were borrowed from the TOEFL vocabulary questions. We prepared three sets of distractors for each question: one generated by the baseline, one generated by the proposed method and one from the original TOEFL question.

In the test taker-based evaluation, we administered the generated questions to 80 English learners and analysed the quality of the distractors based on their responses. We calculated the correlation between the test taker’s scores on the automatically generated questions and their scores on the original TOEFL questions as well as the correlation with their TOEIC scores. As a result, the scores on the questions prepared using the proposed method correlate better with those of the original TOEFL questions than those using the baseline. However, there is no difference between them with respect to the correlation with the TOEIC scores. The scores on the original TOEFL questions showed the highest correlation with those of the TOEIC scores.

We further analysed the characteristics of the distractors using Neural Test Theory. The result showed that the proposed method produced fewer problematic MI distractors than the baseline.

The original TOEFL questions produced the least number of MI distractors. This is encouraging because the proposed method succeeded in removing problematic distractor candidates during their generation better than the baseline.

In the expert-based evaluation, we asked a human expert to judge the quality of the three sets of distractors on a scale from 1 (low quality) to 5 (high quality). The average scores indicate that the distractors generated by the proposed method are better in quality than those generated by the baseline and are comparable in quality with the original distractors.

Further analysis showed that 60% of the baseline problematic distractors from the test taker-based evaluation were also considered problematic by the human expert. In contrast, only 35% and 21% problematic distractors from the proposed method and the original TOEFL questions, respectively, were judged as problematic by the human expert. This is an encouraging result because these distractors can be used for a real test despite their low score from the human expert.

Although the proposed method removes synonyms of the target word and correct answer from the distractor candidates, the expert-based evaluation showed that it still produces problematic distractors that are too similar to the correct answer. The distractors of this category can make a generated question invalid because it appears to have multiple correct answers. The questions generated by the proposed method still need human validation before using for a real test.

Future work for this research includes improving the automatically generated distractors so that they are as close as possible in quality to the human-made ones, especially with respect to their distractive power and plausibility.

## Footnotes

1
http://lang-8.com

2 They used about 14 million sentences from Chinese Wikipedia.

3 Available at https://github.com/idio/wiki2vec/, the model consists of 1000 dimensions, 10 skipgrams and no stemming.

4 In their paper, they use the term “carrier sentence” because the text is usually only a sentence, not necessarily a reading passage as in the present study.

5 Available as a package in the Natural Language Toolkit (NLTK).

6
http://www.natcorp.ox.ac.uk/

7 The word vectors were trained on Wikipedia 2014+ Gigaword 5, which consists of 6B tokens 400K vocabulary uncased words and provides 100 dimensional vectors. This resource is available at https://nlp.stanford.edu/projects/glove/

8
http://www.nltk.org/_modules/nltk/collocations.html

9 Corpora: Brown, ABC, Genesis, Web Text, Inaugural, Gutenberg, Treebank and Movie Reviews, available at www.nltk.org/nltk_data/

10
www.ets.org

11 The Official Guide to the New TOEFL iBT, 2007, published by McGraw-Hill, New York.

12 Oxford Dictionary of English (www.oxforddictionaries.com) and the Merriam-Webster Dictionary (www.dictionaryapi.com).

13 The Merriam-Webster Dictionary’s related-word feature.
